# An elegant route to overcome fundamentally-limited light extraction in AlGaN deep-ultraviolet light-emitting diodes: Preferential outcoupling of strong in-plane emission

**DOI:** 10.1038/srep22537

**Published:** 2016-03-03

**Authors:** Jong Won Lee, Dong Yeong Kim, Jun Hyuk Park, E. Fred Schubert, Jungsub Kim, Jinsub Lee, Yong-Il Kim, Youngsoo Park, Jong Kyu Kim

**Affiliations:** 1 Department of Materials Science and Engineering, Pohang University of Science and Technology, Pohang, 790-784, Korea; 2 Future Chips Constellation, Department of Electrical, Computer, and Systems Engineering, Rensselaer Polytechnic Institute, Troy, NY 12180 USA; 3 Advanced Development Team, LED Business, Samsung Electronics, Yongin 446-920, Korea

## Abstract

While there is an urgent need for semiconductor-based efficient deep ultraviolet (DUV) sources, the efficiency of AlGaN DUV light-emitting diodes (LEDs) remains very low because the extraction of DUV photons is significantly limited by intrinsic material properties of AlGaN. Here, we present an elegant approach based on a DUV LED having multiple mesa stripes whose inclined sidewalls are covered by a MgF_2_/Al omni-directional mirror to take advantage of the strongly anisotropic transverse-magnetic polarized emission pattern of AlGaN quantum wells. The sidewall-emission-enhanced DUV LED breaks through the fundamental limitations caused by the intrinsic properties of AlGaN, thus shows a remarkable improvement in light extraction as well as operating voltage. Furthermore, an analytic model is developed to understand and precisely estimate the extraction of DUV photons from AlGaN DUV LEDs, and hence to provide promising routes for maximizing the power conversion efficiency.

Al_*x*_Ga_1–*x*_N-based deep ultraviolet (DUV) light-emitting diodes (LEDs) emitting at wavelengths as short as 210 nm (AlN) have attracted considerable attention for a variety of applications such as air and water purification, food sterilization, UV curing, medical diagnostics, and defense applications^1^,^2^,^3^,^4^,^5^,^6^,^7^. However, the external quantum efficiency (EQE) of Al_*x*_Ga_1–*x*_N DUV LEDs decreases rapidly as the Al molar fraction *x* increases mainly due to intrinsic material properties of Al_*x*_Ga_1–*x*_N causing inherently poor light-extraction efficiency (LEE)^2^,^3^,^8^. Focused research on efficient DUV LEDs have recently led to remarkable improvements including 10.4% EQE for 278 nm AlGaN DUV LED^8^; nevertheless, typical EQE values of DUV LEDs are less than 5% and decrease to 10^−6^% for 210 nm AlN LEDs^9^.

There are intrinsic material properties of Al_*x*_Ga_1–*x*_N which fundamentally limit the LEE. Firstly, DUV light emitted by a high Al-content Al_*x*_Ga_1–*x*_N multi-quantum well (MQW) active region grown on a *c*-plane sapphire substrate is strongly transverse-magnetic (TM) polarized anisotropic emission originating from the crystal-field split-off hole band being the top-most valence band^10^,^11^,^12^,^13^,^14^. Secondly, the acceptor (*E*_A_) and donor (*E*_D_) activation energies increase with increasing Al molar fraction (*E*_A_ = 170 meV, *E*_D_ = 15 meV for GaN and *E*_A_ = 630 meV, *E*_D_ = 62 meV for AlN)^15^,^16^, resulting in highly resistive p- and n-AlGaN layers. In particular, the hole concentration of p-Al_*x*_Ga_1–*x*_N (*x* > 0.5) is too low to be used as an efficient hole injection and ohmic-contact formation layer. Therefore, typical DUV LEDs have a light-*absorbing* p-GaN layer on top of the p-AlGaN layer thereby abandoning almost half of the DUV emission, which forces the adoption of a bottom-emitting (substrate-emitting) flip-chip configuration. Meanwhile, the highly resistive n-AlGaN causes a localized emission near the edge of the active mesa, referred to as the current crowding effect, strongly affecting the LEE. Consequently, conventional LEE-enhancing techniques such as surface texturing^17^,^18^, substrate patterning^19^, anti-reflective coatings^20^, highly reflective mirrors on top of the p-(Al)GaN^21^ and on the inclined sidewalls along the edge of the square-shape active mesa^22^,^23^,^24^,^25^, all of which favor extracting transverse electric (TE) polarized light, turn out to be much less effective for AlGaN DUV LEDs^8^,^26^. Although a top-emitting LED design reflecting the TM polarized emission toward the p-GaN direction by using Al-coated regrown GaN has been demonstrated^27^, it is ineffective for the bottom-emitting flip-chip configuration necessary for outcoupling DUV emission towards the sapphire substrate, as well as for effective heat dissipation needed for high-power operation and long lifetime^28^. Accordingly, this calls for a totally new approach overcoming the fundamental LEE limitation in DUV LEDs.

In this study, we present a bottom-emitting sidewall-emission-enhanced (SEE) DUV LED having multiple mesa stripes with inclined sidewalls covered by a MgF_2_/Al omni-directional reflector. We show that the SEE DUV LEDs show remarkable improvement in light extraction, enabled by reflecting the strong in-plane emission towards the sapphire substrate, as well as reduced operating voltage. In addition, an analytic model is proposed to precisely estimate the LEE, thus providing viable routes for maximizing the EQE of AlGaN DUV LEDs by taking advantage of the very material properties of AlGaN that, paradoxically, cause the fundamental limitations in the first place.

## Results

### Bottom-emitting sidewall-emission-enhanced (SEE) DUV LEDs

**Figure 1:**
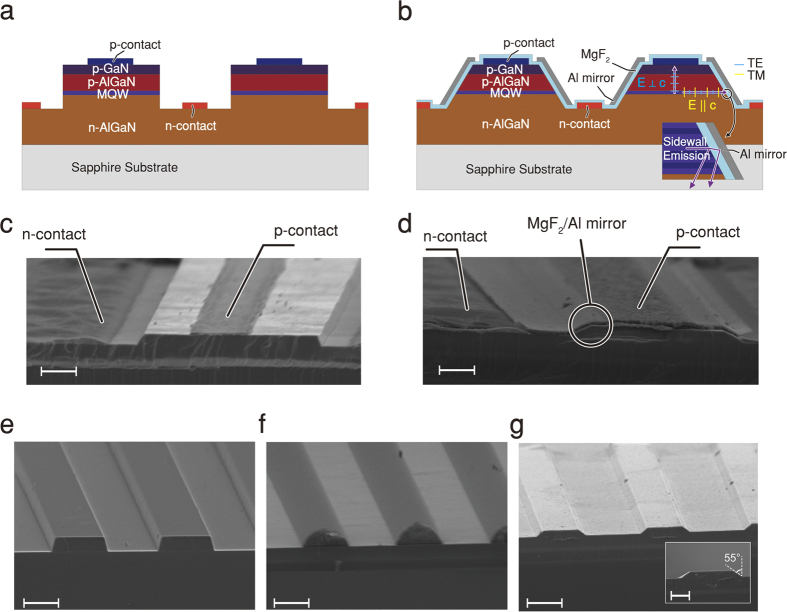
Schematic illustration and scanning electron microscope (SEM) image for reference and SEE DUV LEDs. Schematic illustrations of (**a**) a reference LED with vertical sidewalls and (**b**) a SEE DUV LED with MgF_2_/Al omni-directional reflectors on inclined sidewalls. Each dipole located along the MQW active region emits isotropic TE polarized emission as well as anisotropic TM polarized emission. (**c**) SEM bird’s-eye view of reference LED’s mesa having near-vertical sidewalls, n-contact, and p-contact (scale bar, 2 μm). (**d**) SEM bird’s-eye view of SEE DUV LED. Unlike image c, it shows an Al mirror covering inclined sidewalls (scale bar, 2 μm). (**e**) SEM image of photoresist stripe pattern having vertical sidewalls formed by conventional lithography process (scale bar, 6 μm). (**f**) SEM image of photoresist stripe pattern having a rounded shape after thermal reflow (scale bar, 6 μm). (**g**) SEM image after inductively coupled plasma dry etching and photoresist removal. Inclined sidewalls are formed along the stripes (scale bar, 6 μm). Inset Fig. shows cross-sectional view of the stripe (scale bar, 2 μm).

Figure 1a, b show schematic cross-sectional views of the reference and the bottom-emitting SEE DUV LEDs, respectively. Both LEDs have multiple active mesa stripes in order to expose the sidewall of the MQW active region for effective extraction of the strong TM-polarized sidewall-directed emission. Metal stripe contacts on the n-AlGaN are located between the active mesa stripes. The cross-section of the active mesa stripe for the reference LED is a rectangle, while that for the SEE DUV LED is an isosceles trapezoid with inclined sidewalls coated with an optically transparent dielectric layer followed by Al. The DUV-transparent dielectric layer, MgF_2_, acts as a multifunctional layer; it is an electrical insulating layer between the active region and Al, an electrical passivation layer for the exposed sidewall, and an omni-directional reflector in conjunction with Al^29^ which reflects the strong sidewall-directed TM emission down to the substrate as shown in the inset of Fig. 1b.

Reference and SEE DUV LEDs with 1 × 1 mm^2^ chip area were fabricated by a conventional LED fabrication process. Figure 1c,d are SEM bird’s-eye views of a 45 mesa stripe reference LED and SEE DUV LED, respectively. The inclined sidewalls of the mesa stripes, coated with MgF_2_/Al omni-directional reflectors, for the SEE DUV LED were fabricated by photoresist patterning using conventional lithography (Fig. 1e), reflow of photoresist (Fig. 1f), and dry etching (Fig. 1g). The detailed fabrication processes and the fabricated devices having various numbers of stripes (from 5 to 50) are shown in Supplementary Figs. S1 and S2, respectively. Note that as the number of active mesa stripes increases while maintaining a fixed chip area, the perimeter length of the exposed sidewall increases while the area of the active mesa decreases, as summarized in Supplementary Table S1.

Electroluminescence spectra and the TE/TM polarization ratio were measured at various injection currents. The DUV LED’s peak emission wavelength is 275 nm. The intensity increases linearly upon increasing the injection current. The degree of polarization defined as 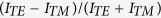 was measured to be −0.021 (Supplementary Fig. S3), consistent with results reported in the literature^30^, indicating that more than half of the emission is TM polarized. The MgF_2_/Al mirror formed on the inclined sidewalls with angle of ~35° shows a high reflectivity for DUV photons and omni-directional characteristics. Experimental and calculated optical properties of the MgF_2_/Al reflector are described in Supplementary Fig. S4.

### Ray tracing simulations

**Figure 2:**
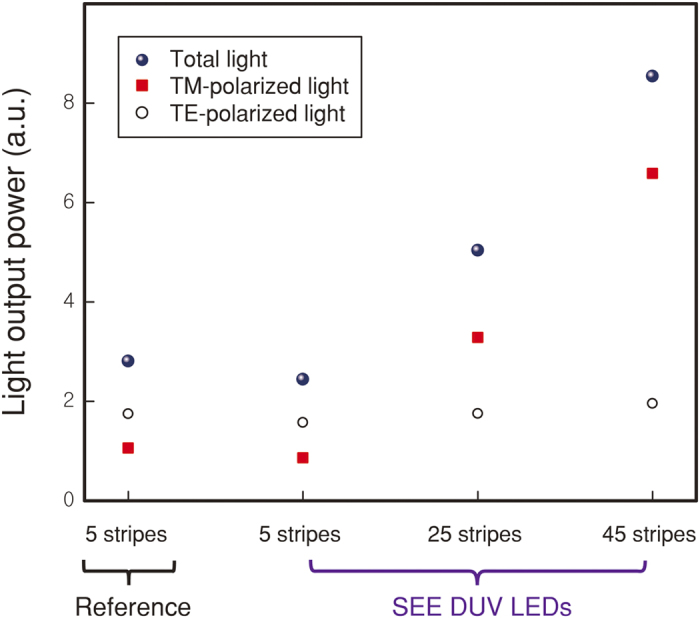
Ray tracing results. Calculated light output power from the reference LED with 5 stripes having vertical sidewalls and from the SEE DUV LEDs with 5, 25, 45 stripes having MgF_2_/Al reflectors on inclined sidewalls. The SEE DUV LED shows a remarkable increase of the total light output power as the number of stripe increases.

Ray tracing simulations were performed for various DUV LED structures to check the feasibility of the SEE DUV LED concept. Light output power from the reference LED with 5 stripes having vertical sidewalls and from the SEE DUV LEDs with 5, 25, 45 stripes having MgF_2_/Al reflectors on inclined sidewalls were simulated. The epitaxial structure and the geometrical parameters of simulated LEDs are the same as for the actually fabricated LEDs (See Supplementary Fig. S5 and Supplementary Table S2 for detailed information on the ray tracing simulations). Figure 2 shows the simulated light output power of both TE and TM polarized light extracted through the sapphire substrate. Both TE and TM polarized emissions are enhanced as the number of stripes increases. In particular, anisotropic TM polarized light shows a much steeper enhancement than isotropic TE polarized light with increasing number of stripes. This is because more anisotropic TM polarized light is reflected at the sidewall reflectors than isotropic TE light, leading the remarkable increase of the total light output power as the number of stripe increases. Ray tracing simulation results indicate that utilizing the strong sidewall emission is essential to enhancing the LEE in AlGaN based DUV LEDs, consistent with the numerical analyses performed by the finite element method. (See Supplementary Fig. S6 for detailed information on the finite element method simulations and additional results).

### Performance of SEE DUV LEDs

**Figure 3:**
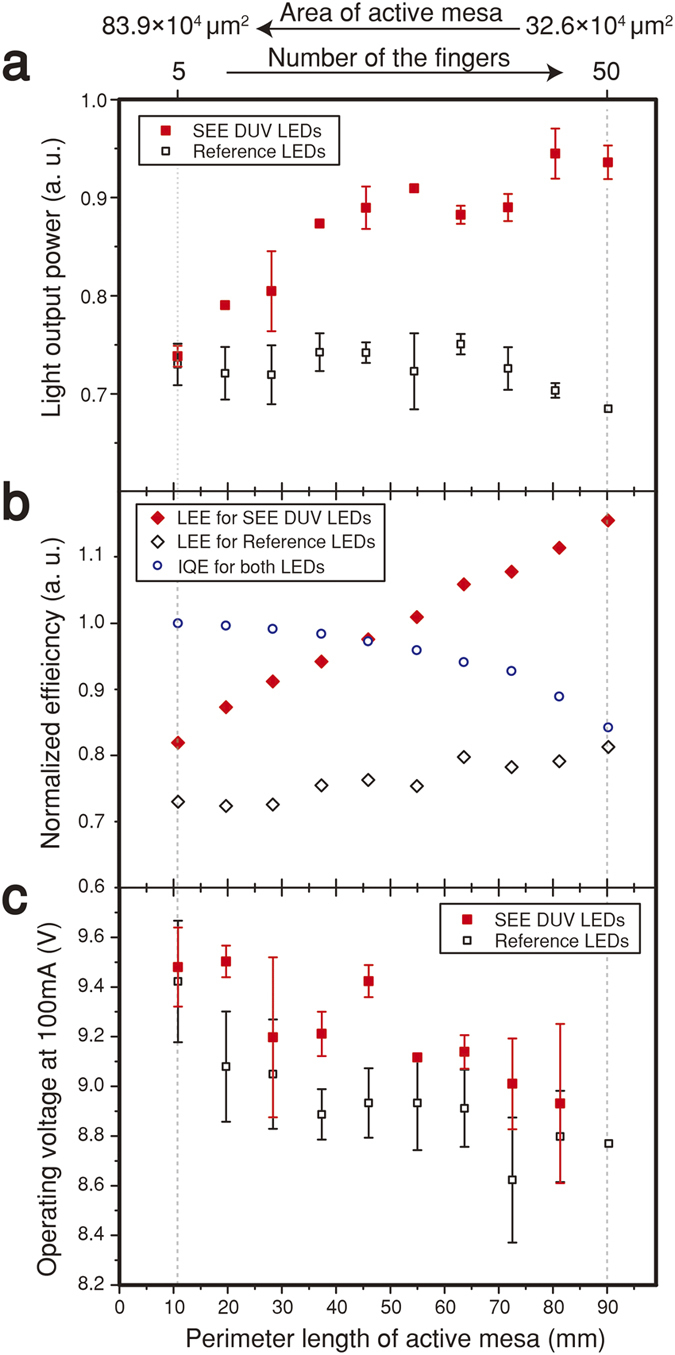
Measured light output power, inferred light extraction efficiency (LEE), and operating voltage at 100 mA as a function of perimeter length of active mesa. (**a**) Measured light output power for the reference and SEE DUV LEDs. The increase in perimeter length of the active mesa is proportional to the increase in the number of the fingers (ranging from 5 to 50) and decrease in area of the active mesa (from 83.9 × 10^4^ to 32.6 × 10^4^ μm^2^). (**b**) LEE inferred from measured internal quantum efficiency and external quantum efficiency. Relative values of IQE and EQE are obtained from the representative EQE curve and light output power values. (**c**) Operating voltage at drive current of 100 mA for SEE DUV LED and reference LED. Operating voltages at 100 mA drive current are obtained from the IV curves.

Figure 3a shows light output power (LOP) of the reference and the SEE DUV LEDs at the drive current of 100 mA as a function of the perimeter length of the active mesa which is proportional to the number of the stripes ranging from 5 to 50 and the associated reduction in active-mesa area from 83.9 × 10^4^ to 32.6 × 10^4^ μm^2^, respectively. The LOP of the SEE DUV LEDs increases as the perimeter length of the active mesa increases, and seems to saturate at perimeter lengths longer than ~60 mm, whereas the reference LEDs initially show very little variation of the LOP followed by a slight decrease. As the perimeter length of the active mesa increases at a given drive current, the sidewall-directed emission and current density increase as well. Since the SEE DUV LEDs utilize the sidewall emission much more effectively than the reference LEDs, the former show a linear increase in LOP upon increasing the perimeter length while the latter do not show a notable increase. As the perimeter length further increases, the efficiency droop^31^–the decrease of internal quantum efficiency (IQE) with increasing injection current *density*–becomes more significant, causing a saturation in LOP for the SEE DUV LEDs, and a decrease in LOP for the reference LEDs. In addition, the current crowding effect near the sidewall of the active mesa also affects the variation of the LOP when increasing the perimeter length.

Figure 3b shows the normalized IQE and relative LEE of the reference LED and the SEE DUV LED as estimated from the measured LOP shown in Fig. 3a (See Supplementary Fig. S8 for details of the estimation). At a fixed drive current, 100 mA in this study, the drive current density varies with the area of the active region. The smaller the active region area, i.e., the higher the current density, the more the IQE is reduced due to the efficiency droop. Despite the decrease in IQE, the EQE increases upon increasing the perimeter length due to the remarkable enhancement in LEE enabled by the MgF_2_/Al mirror that reflects the strong sidewall-directed emission. The maximum enhancement in LEE of 58.3% is obtained for the SEE DUV LED with 50 stripes when compared to the reference LED with 5 stripes.

Figure 3c shows the variation of the operating voltage at a drive current of 100 mA for the reference and the SEE DUV LEDs as a function of the perimeter length of the active mesa. As the perimeter length increases, the operating voltage decreases for both types of LEDs, which is attributed to a reduced ohmic contact resistance for the larger n-AlGaN contact area for the LEDs with a longer perimeter length (See Supplementary Fig. S7 for representative current-voltage characteristics). Note that the enhanced LOP in conjunction with the reduced operating voltage for the SEE DUV LEDs with a long perimeter length should result in a remarkable improvement in the power efficiency–also called the *wallplug* efficiency–defined as the ratio of the LOP to the input electrical power (current times operating voltage).

### Analytic modeling for LEE estimation

**Figure 4:**
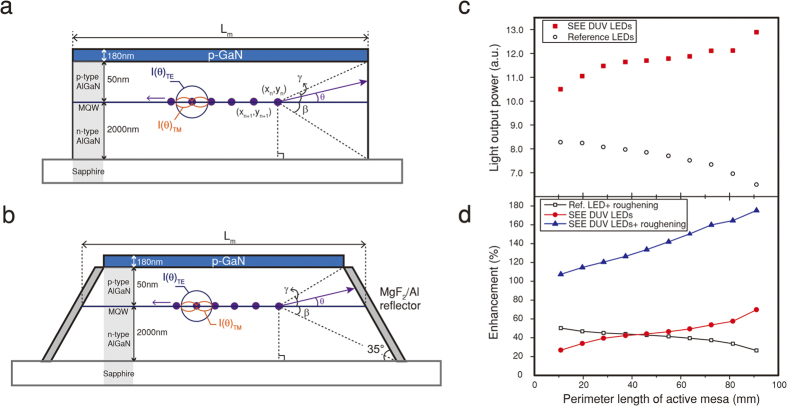
Analytic modeling of light extraction in SEE DUV LEDs. (**a**) Schematic illustration of the reference structure having vertical sidewall. Dipole sources are located between the p-AlGaN and n-AlGaN layer and emit 275 nm TE polarized light and TM polarized light. (**b**) Schematic illustration of SEE DUV LEDs having inclined sidewall with omni-directional reflector. Deep UV photons heading for the p-GaN layer and heading for the reflector are mostly absorbed and mostly reflected, respectively. (**c**) Simulated LOP for various perimeter lengths of active mesa. (**d**) Enhancement ratio of reference LED with sapphire roughening, SEE DUV LEDs, and SEE DUV LEDs with sapphire roughening, relative to the reference LED with no stripe and no roughening.

In order to understand the enhanced LEE of the SEE DUV LEDs and other critical factors affecting the LEE and EQE of DUV LEDs, we propose an analytic model to estimate the LEE. Figure 4a,b show the cross-sections of active mesa stripes of the reference LED and the SEE DUV LED, respectively, consisting of a p-GaN contact layer, a p-AlGaN layer, and an n-AlGaN layer on a sapphire substrate. For the SEE DUV LED, the MgF_2_/Al omni-directional reflector is placed along the inclined sidewalls of the active mesa stripes. *N* dipole sources are located equi-distantly between the p-AlGaN and the n-AlGaN where the MQW active region is located. The polarization ratio of the emitted light can be adjusted depending on the Al molar fraction in AlGaN; corresponding TE and TM polarized light intensities are described by 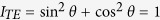 and 
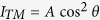
, respectively^32^, where 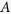 is a parameter defined as the intensity ratio of polarized light 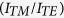 travelling along the in-plane direction (“*A* parameter”). For the SEE DUV LED used in this study, 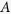 is set to 1 based on the measured polarization ratio shown in Supplementary Fig. S3b. For an accurate estimation of extracted light from each dipole source through the bottom side, several factors should be considered including the location of *n*^th^ dipole source (*x*_*n*_, *y*_*n*_) and its traveling length *l(n*, θ), absorption coefficient (α)^33^, refractive index of each layer, and the width of the stripe *L*_*m*_ determined by the number of stripes *m*. In particular, the current crowding effect^34^ should be taken into account due to the lateral current transport in AlGaN DUV LEDs with a resistive n-AlGaN layer, which is considered by introducing the following weighting factor 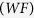 for n^th^ dipole source with the current spreading length (*L*_s_) expressed as,


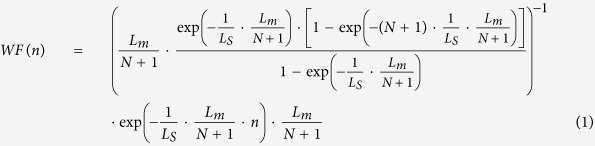


In addition, the effect of texturing or roughening of the sapphire/AlGaN interface and sapphire substrate on LEE is considered by introducing the *effective* critical angle θ_c_^*^ determined by the optical properties at the interface between two optical media, for example the sapphire/air interface.

The LOP of extracted light through the sapphire substrate is the sum of the LOP from *n*^th^ dipole source directly impinging down to the bottom substrate (*LOP*_*m,direct*_), toward the top p-GaN, and reflected by the omni-directional reflector with reflectivity *R* (*LOP*_*m,refelcted*_). Assuming that the light emitted toward the p-GaN is totally absorbed, the total LOP can be expressed as,


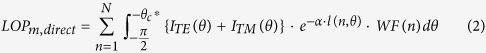



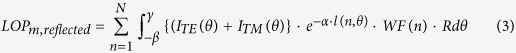



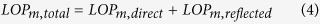


Detailed information on the integration range, the effective critical angle, and weighting factor associated with current crowding is also provided (See Supplementary Information).

Figure 4c shows the total LOP determined by using the analytic model. The LOP of the SEE DUV LED increases as the perimeter length of the active mesa increases, whereas the reference LEDs show a very slight decrease. It is found that *LOP*_*m,direct*_ is mostly composed of TE polarized light for both LEDs, and is slightly decreased due to the efficiency droop effect at high current densities. On the other hand, *LOP*_*m,reflected*_ is composed of both TE and TM polarized light and is greatly enhanced only for the SEE DUV LEDs as the perimeter length of the active mesa increases due to larger MgF_2_/Al reflector area. Note that the simulated LOP value and trend is very similar to the experimental data and to the estimated LEE shown in Fig. 3a,b, respectively, confirming the validity of the model.

One of widely-used ways to enhance the LEE of InGaN LED is roughening the backside of the sapphire substrate to overcome total internal reflection. By using the analytic model, the effect of sapphire roughening on LEE for both reference and the SEE DUV LEDs were calculated in terms of the enhancement ratio defined as (*LOP*_LEE-enhancing technique_–*LOP*_reference_)/*LOP*_reference_. Figure 4d shows the LEE enhancement for the SEE DUV LED, for the reference LED with roughened substrate, and for the SEE DUV LEDs with roughened substrate. Roughening the sapphire substrate enlarges the effective critical angle (i.e. the escape cone), and thus the integration range for *LOP*_*m, direct*_. As discussed, the enhancement ratio for the SEE DUV LED increases by up to ~70% as the perimeter length of the active mesa increases. The LEE enhancement ratio (due to roughening) of the reference LED to that of the SEE DUV LED decreases as the perimeter length increases, showing the effectiveness of the SEE concept especially for longer perimeter lengths and strongly TM polarized light. Roughening the sapphire substrate of the SEE DUV LED results in a remarkable enhancement up to 175%, which is directly attributed to TM and TE polarized light being efficiently reflected by the sidewall mirror and extracted by the larger escape cone, respectively.

### Future perspectives

Based on the very promising experimental results from the SEE DUV LEDs as well as the proposed analytic model, viable routes to further enhance the EQE by utilizing the intrinsic material properties of AlGaN are discussed. Firstly, as shown in Fig. 4d, the concept of the SEE DUV LED becomes even more powerful when it is synchronized with other LEE-enhancing techniques such as surface texturing, substrate patterning, anti-reflective coatings, and highly reflective mirrors, resulting in remarkable synergistic effects.

**Figure 5:**
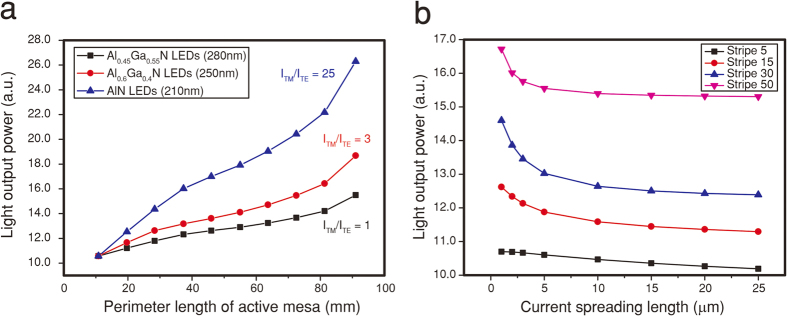
LOP expectation for SEE deeper UV LEDs (λ ≤ 280 nm) with changing mesa perimeter length and current spreading length. (**a**) Light output power as a function of the mesa perimeter length for various *A* parameters (*A* = *I*_TM_/*I*_TE_). As the emission wavelength decreases, TM-polarized light becomes dominant, meaning the *A* parameter increases. That is, more light can be extracted for a large *A*-parameter condition. (**b**) Light output power for various stripe structures as a function of the current spreading length. For smaller current spreading lengths, the SEE concept is more effective.

Secondly, we note that the SEE DUV LED concept will be even more effective for *deeper* UV LEDs (λ ≤ 280 nm). As the Al molar fraction increases in Al_*x*_Ga_1–*x*_N, TM polarized emission becomes much stronger, i.e., giving a higher *A* parameter 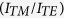, and current crowding becomes much more severe, resulting in poor LEE. Figure 5a shows the LOP, calculated by the analytic model, for various 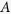 parameters 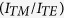 as a function of the mesa perimeter length. 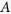 = 1 and 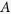 = 3 correspond to a 280 nm Al_0.45_Ga_0.55_N LED and a 250 nm Al_0.6_Ga_0.4_N LED^35^, respectively. As an extreme case, a 210 nm AlN LED having 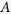 = 25 is calculated^12^ and shows the greatest advantage. The LOPs are normalized to the LOP of the reference LED without a stripe. As the mesa perimeter length increases, the calculated LOP increases for all cases, consistent with experimental results. As the 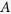 parameter increases with Al molar fraction, the amount of sidewall-reflected light increases, resulting in much more extracted light, indicating that the SEE concept is even more effective as the emission wavelength decreases.

In addition, the current spreading length (*L*_s_), which is determined by the resistivity of n-AlGaN layers, becomes increasingly important in deeper UV LEDs. The current spreading length for conventional 280 nm emitting DUV LED^36^ is estimated to ~20 μm and decreases for shorter emission wavelengths. Figure 5b shows the LOP of SEE DUV LEDs with various numbers of stripes as a function of the current spreading length, As the current spreading length decreases due to more resistive n-AlGaN, radiative recombination occurs increasingly near edge of the mesa structure where light is effectively reflected by the sidewall reflector further illustrating the advantage of the SEE concept.

In conclusion, we presented bottom-emitting (substrate-emitting) AlGaN SEE DUV LEDs that take advantage of the strongly TM polarized emission. Consistent with the ray tracing simulation results, both optical (LOP) and electrical (operation voltage) properties of the SEE DUV LEDs are improved as the perimeter length of the active mesa increases, which is enabled by an effective utilization of the anisotropic sidewall-directed emission, showing the validity and potential of the SEE concept. Furthermore, an analytic model is developed to understand the enhanced LEE, and hence, to propose viable routes to further enhance the LEE. It was shown that SEE concept allows for a remarkable synergistic effect when combined with the conventional LEE-enhancing techniques (such as substrate surface roughening), and becomes much more effective for deeper UV LEDs (< 275 nm).

## Methods

### Growth and device fabrication

The DUV LED structures (λ_peak_ = 275 nm DUV emission) were grown on (0001) 4-inch diameter sapphire substrates by metal-organic vapor phase epitaxy. A low-temperature AlN layer and AlGaN/AlN superlattice layer serve as buffer layers for the subsequent growth of the active layers including a 2 μm Si doped ([Si] = 1 × 10^18^ cm^−3^) n-Al_0.55_Ga_0.45_N, five periods of multiple quantum wells (MQWs) composed of undoped 1.5 nm-thick Al_0.43_Ga_0.57_N wells and 10 nm-thick Al_0.5_Ga_0.5_N barriers. Finally, a 15 nm-thick Mg-doped ([Mg] = 2 × 10^18^ cm^−3^) high-Al-content AlGaN electron-blocking layer, a 2 nm-thick Mg-doped ([Mg] = 2 × 10^18^ cm^−3^) p-Al_53_Ga_47_N layer, a 15 nm-thick p-AlGaN cladding layer having Al composition grading from 53% to 0%, a 160 nm-thick highly Mg doped ([Mg] > 2 × 10^20^ cm^−3^) p-GaN layer, and a 20 nm-thick highly Mg doped ([Mg] > 4 × 10^20^ cm^−3^) p^ + ^-GaN thin contact layer were grown.

The reference and the SEE DUV LEDs with various numbers of stripes (from 5 to 50) formed on 1 × 1 mm^2^ chip area were fabricated. First, a photoresist was spun on the DUV LED epitaxial structure and active mesa stripes were patterned by conventional photolithography. In order to obtain an isosceles trapezoidal active mesa structure with inclined sidewalls, we utilized thermal reflow of photoresist to form a hemisphere shape, as commonly used in making PSS (patterned sapphire substrates), followed by inductively coupled plasma dry etching down to ~1 μm to expose the n-AlGaN. Then, metal layers Ti/Al/Ni/Au (30/120/40/100 nm) were deposited on the exposed n-AlGaN surface by electron-beam/thermal evaporation, followed by annealing at 900 °C for 1 min in N_2_ ambient to form an ohmic contact. The p-contact Ni/Au (20/100 nm) was also deposited with the same method and annealed at 750 °C for 1 min in air ambient. Then, Ti/Au (20/100 nm) pad metals were formed on both n- and p-type contacts. MgF_2_/Al (250/150 nm) omnidirectional reflectors were formed on the inclined sidewalls of the active mesa stripes. Please see Supplementary Figs. S1 and S2 for details of the device fabrication.

### Measurements

The light output at 100 mA, 5 ms current pulse (1% duty cycle) was measured as a photocurrent using a Si photodetector, an Agilent B2902A Precision Source/Measurement unit, using a bottom-emission measurement setup with the samples (processed epi-wafer pieces) in a freestanding condition in a dark room environment. The distance between sample and detector is 4 cm; thus detected light emission can be considered as a far field emission. The Detector can be rotated from the bottom (substrate) to the top (p-GaN) while maintaining the same distance to the sample; this allows for the light emission pattern to be measured. Electroluminescence spectra were taken using a UV-VIS spectrometer (Black C-50, StellarNet Inc.) and UV-enhanced optical fiber (F1000-UV-VIS-SR) at various injection currents. For the polarization measurement, Glan-Taylor polarizer (SM05PM5, Thorlabs) is applied to the EL measurement setup and the light output power is measured at the horizontal direction (See supplementary Fig. 3b). IV characteristics are measured from −5 V to 15 V, under DC current condition using Agilent B2902A Precision Source/Measurement Unit.

## Additional Information

**How to cite this article**: Lee, J. W. *et al*. An elegant route to overcome fundamentally-limited light extraction in AlGaN deep-ultraviolet light-emitting diodes: Preferential outcoupling of strong in-plane emission. *Sci. Rep.* **6**, 22537; doi: 10.1038/srep22537 (2016).

## Supplementary Material

Supplementary Information
