# Supplementary material for: An elegant route to overcome fundamentally-limited light extraction in AlGaN deep-ultraviolet light-emitting diodes: Preferential outcoupling of strong in-plane emission
Source: Sci Rep. 2016 Mar 3;6:22537. doi: 10.1038/srep22537 (PMC4776099; doi:10.1038/srep22537)
Supplement: Supplementary Information [file srep22537-s1.pdf]

Supplementary Information

An elegant route to overcome fundamentally-limited light extraction in AlGaN deep-ultraviolet light-emitting diodes: Preferential outcoupling of strong in-plane emission

Jong Won Lee[1], Dong Yeong Kim[1], Jun Hyuk Park[1], E. Fred Schubert[2],

Jungsub Kim[3], Jinsub Lee[3], Yong-Il Kim[3], Youngsoo Park[3], and Jong Kyu Kim[1*]

[1]Department of Materials Science and Engineering, Pohang University of Science and Technology, Pohang, 790-784, Korea

[2]Future Chips Constellation, Department of Electrical, Computer, and Systems, Engineering, Rensselaer Polytechnic Institute, Troy, NY 12180 USA

[3]LED Business, Samsung Electronics, Yongin 446-920, Korea

*Correspondence and requests for materials should be addressed to J.K.K. (e-mail: kimjk@postech.ac.kr)

Supplementary Figure S1. Fabrication processes for SEE DUV LEDs

Supplementary Figure S1 shows the fabrication steps for a SEE DUV LED with inclined and reflective sidewalls of the active mesa. Figure S1a shows the schematic epitaxial structure of an AlGaN-based DUV LED grown on a 4 inch sapphire substrate. First, the active mesa stripes were patterned by a conventional photolithography (Fig. S1b). In order to obtain the active mesa stripes with inclined sidewalls, thermal reflow of photoresist was carried out at 140 ℃ for 10 minutes to form a rounded shape (Fig. S1c), followed by inductively coupled plasma dry etching to expose the n-AlGaN (Fig. S1d). Then, ohmic contact stripes were patterned on the exposed n-AlGaN between the active mesa stripes, and Ti/Al/Ni/Au (30/120/40/100 nm) were sequentially deposited by using an electron-beam/thermal evaporation, followed by lift-off and annealing at 900 ℃ for 1 min in N_2 ambient to form ohmic contacts. Ni/Au (20/100 nm) ohmic contact stripes were deposited on p-GaN and annealed at 750 ℃ for 1 min in air (Fig. S1e). Then, Ti/Au (20/100 nm) pad metals were formed on both ohmic contact stripes for n-AlGaN and p-GaN. MgF_2/Al (250/150 nm) omni-directional reflectors were formed on the inclined sidewalls of the mesa stripes (Fig. S1f and Fig. S1g) by using conventional photolithography, electron-beam evaporation, and lift-off.

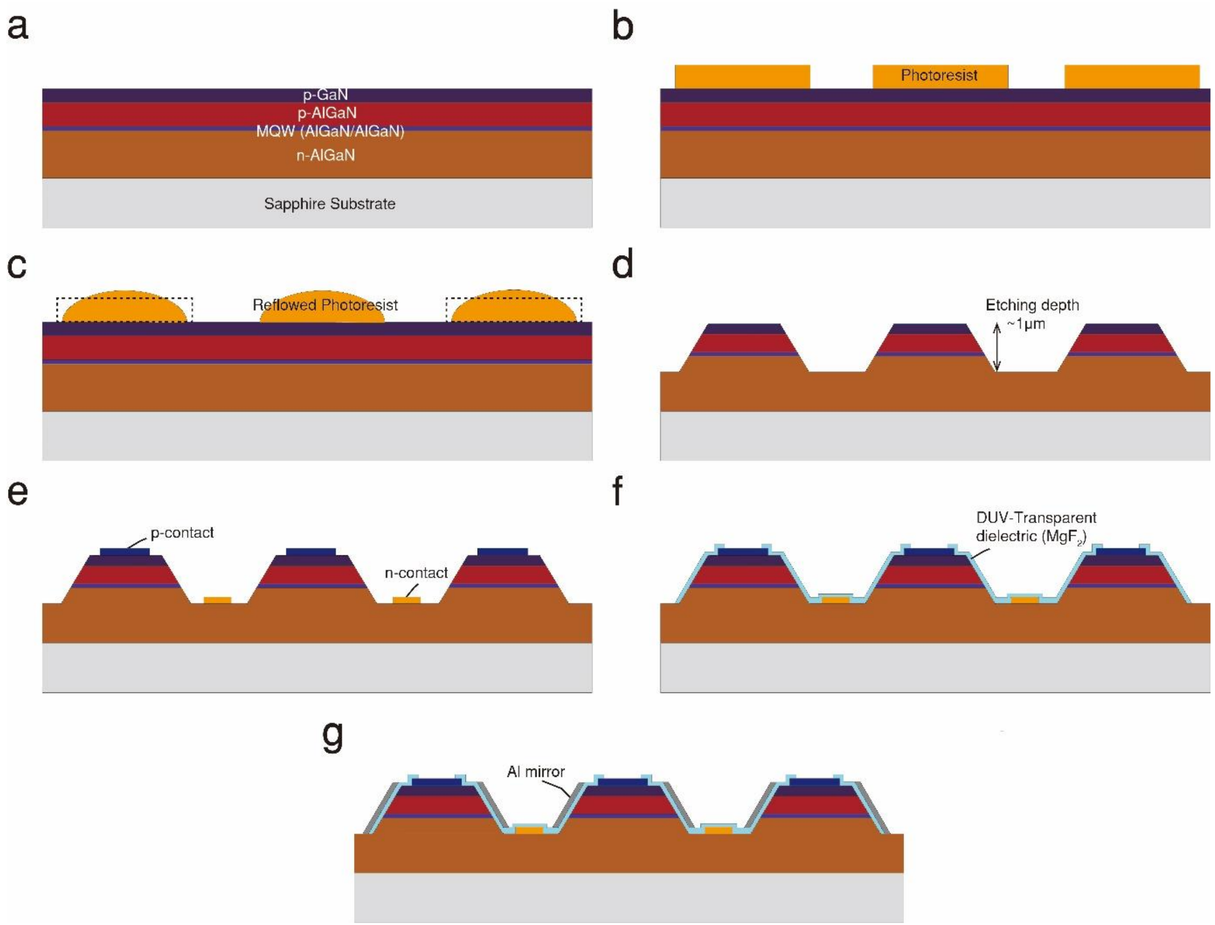

Supplementary Figure S1. Fabrication processes for the SEE DUV LEDs including (a) growth of a DUV LED epitaxial layers on a 4 inch sapphire substrate by metal-organic chemical vapor deposition, (b) patterning of the active mesa, (c) thermal reflow of photoresist to form a rounded etching mask, (d) formation of the active mesa stripe by inductively-coupled plasma etching, (e) ohmic contact formation on both n-AlGaN and p-GaN, (f) deposition of MgF_2 and (g) deposition of Al.

Supplementary Figure S2. SEE DUV LEDs with various numbers of mesa stripes

AlGaN-based SEE DUV LEDs with a 1 × 1 mm^2 chip area and with various numbers of mesa stripes, ranging from 5 to 50, were designed and fabricated. As the number of the stripes increases, the perimeter length of the active mesa increases as summarized in Supplementary Table S1, therefore, the strong TM-polarized sidewall-directed emission can be reflected down to the sapphire substrate by the MgF_2/Al omni-directional mirror that is located on the inclined sidewalls.

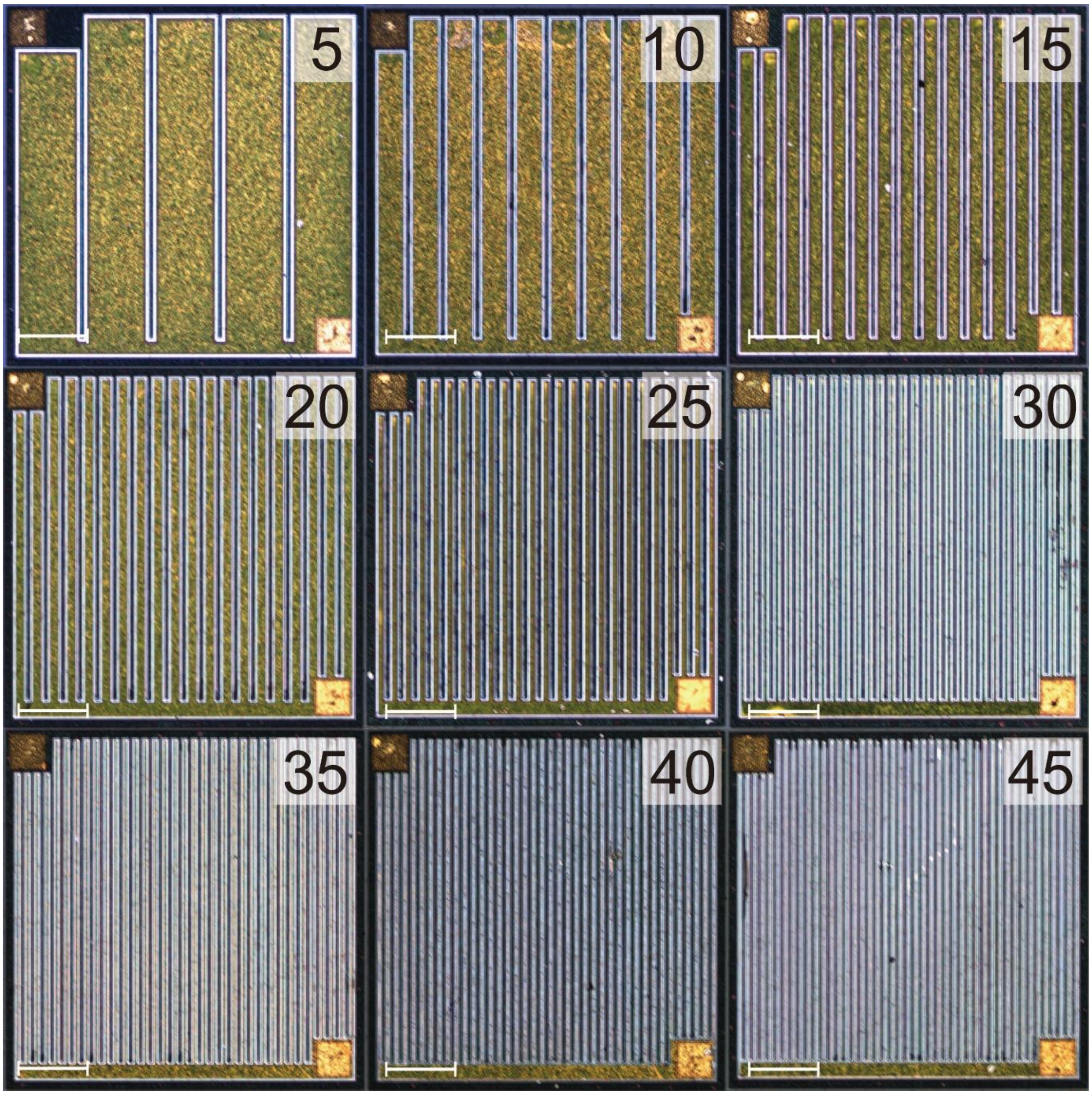

Supplementary Figure S2. Optical microscopy (OM) images of the SEE DUV LEDs with various numbers of the stripes from 5 to 45 (scale bar: 200 μm).

Supplementary Table S1. Perimeter length and active area values for various numbers of stripes

# of Stripes	5	10	15	20	25	30	35	40	45	50
**Perimeter length (mm)**	10.81	19.67	28.34	37.34	46.01	55.01	63.68	72.51	81.35	91.05
**Active area ($\times 10^4$ μm^2)**	83.9	79.0	73.1	67.5	61.7	56.1	50.3	46.7	38.8	32.6

Supplementary Figure S3. EL spectra for various injection currents and TE/TM polarization

The electroluminescence (EL) spectra of a SEE DUV LED with the peak wavelength of 275 nm at various drive currents are shown in Supplementary Fig. S3a. As the drive current increases from 20 mA to 100 mA, the EL intensity linearly increases as well. Supplementary Figure S3b shows the EL spectra at 100 mA along with its TE and TM polarized characteristics. Inset Fig. S3b shows the schematic for the EL and polarization measurement setup. Only a small part of the sample is attached to the double-side-polished sapphire support to make a free standing condition so that DUV absorption by the sapphire support can be neglected. The intensity of each polarized light travelling along the in-plane direction is detected by the StellarNet spectrometer connected with a UV-enhanced optical fiber and Glan-Taylor polarizer. The degree of polarization is defined as,

$$\text{Degree of polarization} = \frac{I_{TE} - I_{TM}}{I_{TM} + I_{TM}}$$

where, I_{TE} and I_{TM} are the intensity of TE- and TM-polarized light, respectively. The polarization ratio of the 275 nm DUV LEDs used in this study was estimated to be -0.021.

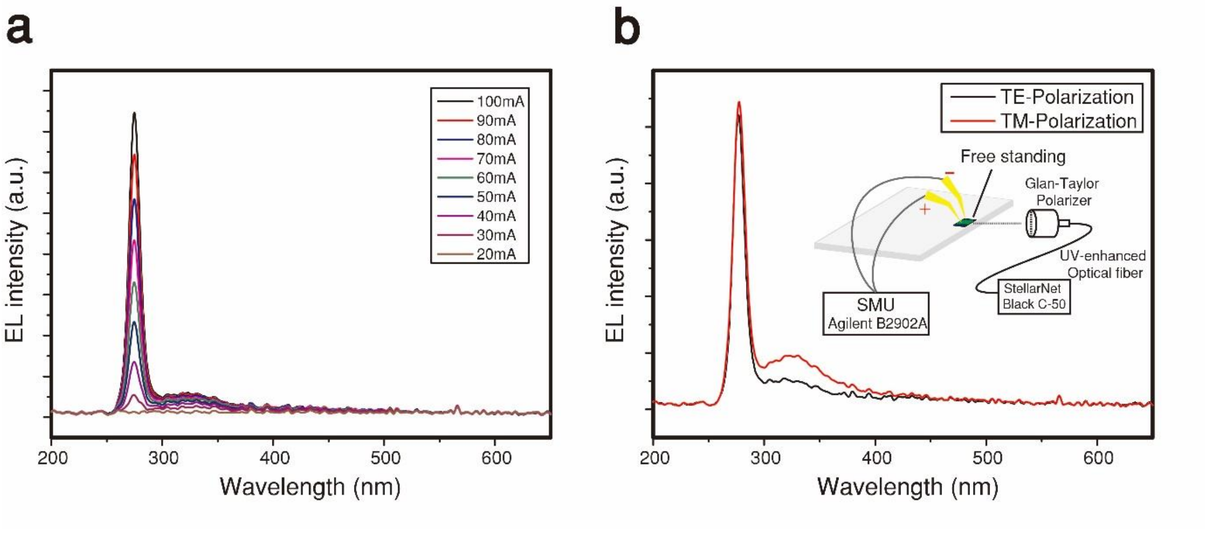

Supplementary Figure S3. (a) Drive current-dependent electroluminescence (EL) spectra of the 275 nm AlGaN-based DUV LED as a function of wavelength. (b) EL spectra of both TE and TM polarized emissions from the 275 nm DUV LED driven at 100 mA.

Supplementary Figure S4. Optical properties of the MgF_2/Al omni-directional reflector (ODR)

Optical characteristics of MgF_2/Al were estimated and experimentally measured. Supplementary Figure S4a shows the calculated reflectance of the MgF_2/Al omni-directional reflector (ODR) at 275 nm as a function of the thickness of MgF_2 layer. Constructive and destructive interferences depend on the thickness of the MgF_2 layer causing the interference fringes. The MgF_2/Al ODR shows a maximum reflectance when the thickness of the MgF_2 layer is 250 nm; this value was chosen for the fabrication of the SEE DUV LEDs. Supplementary Figure S4b shows the calculated reflectance of the ODR as a function of incident angle and the wavelength for the MgF_2 thickness of 250 nm. The ODR shows high reflectance values over a wide range of the incident angle, i.e., it has omni-directional characteristics. Supplementary Figure S4c shows the measured reflectance from the sapphire/AlGaN/MgF_2/Al ODR and the sapphire/AlGaN when 275 nm DUV light impinges with varying incident angle. The ODR shows ~ 30% higher reflectance at all angles than the reference structure, indicating its omni-directional and high-reflectance characteristics. Note that the measured reflectance is lower than the calculated one because it was not measured in an integrating sphere system. Supplementary Figure S4d shows the measured reflectance for the ODR and the reference structure as a function of the wavelength of light with incident angle of 30°, showing that the ODR can effectively reflect the emission wavelength of 275 nm. Both structures show a decrease in reflectance as the emitting wavelength decreases under 270 nm due to large DUV absorption in the AlGaN layer.

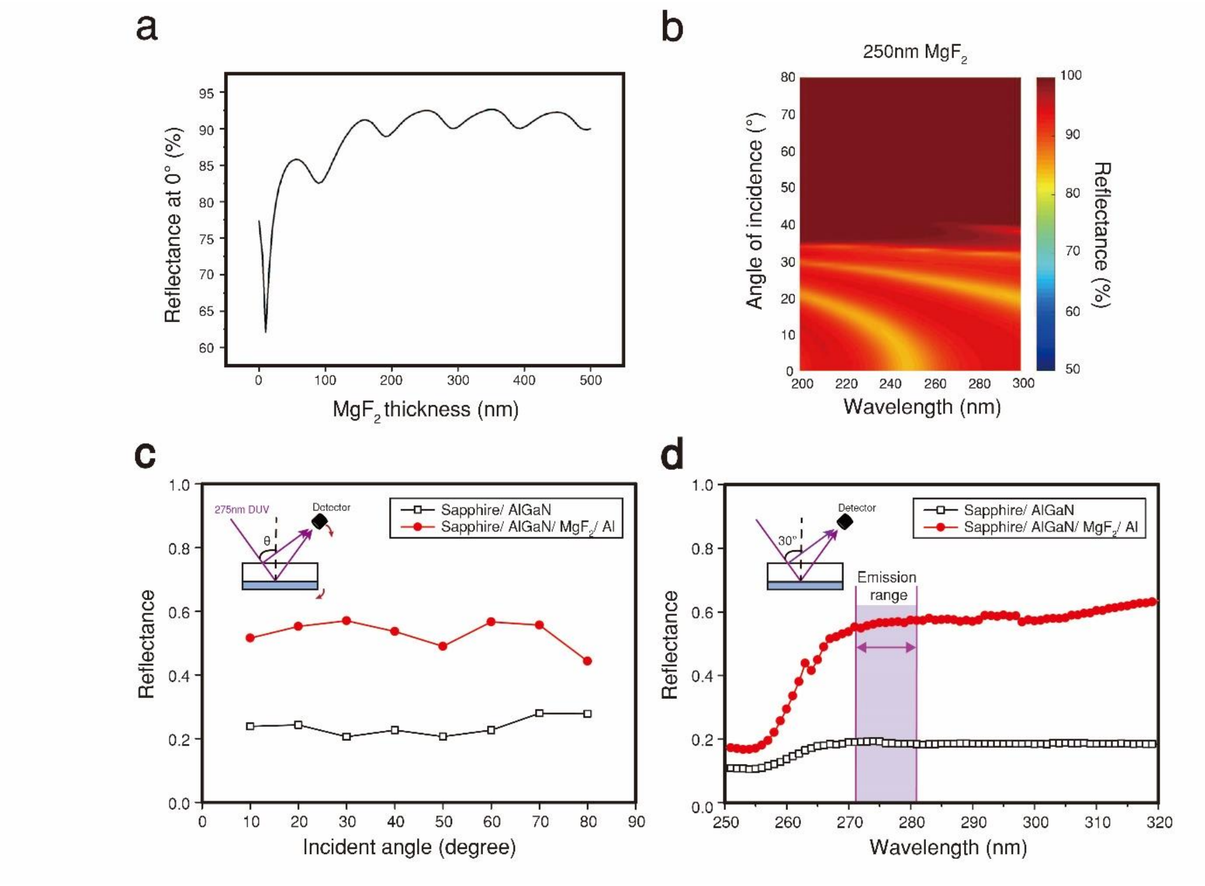

Supplementary Figure S4. (a) Calculated reflectance of the MgF_2/Al ODR as a function of the thickness of the MgF_2 layer. (b) Calculated reflectance contour map as functions of angle and wavelength of the incident light when the thickness of the MgF_2 layer is 250 nm. Measured reflectance of the ODR and the reference structure (c) as a function of angle of incidence for light with 275 nm, and (d) as a function of wavelength with light incident at 30°.

Supplementary Figure S5. Ray tracing simulations

For the ray tracing simulations, absorption coefficients of 10 cm^{-1} and 170,000 cm^{-1} are used for the AlGaN and GaN layer at 275 nm, respectively[1,2]. The refractive indices used for GaN, AlGaN, MgF_2 and Al at 275nm are 2.61, 2.45, 1.4 and 0.216, respectively. The epitaxial structure and the geometrical parameters of simulated LEDs are the same as the actually fabricated LEDs, which are described in the Methods in the manuscript. Total rays of 4×10^6 were simulated in each structures and the light output power was calculated at the bottom side.

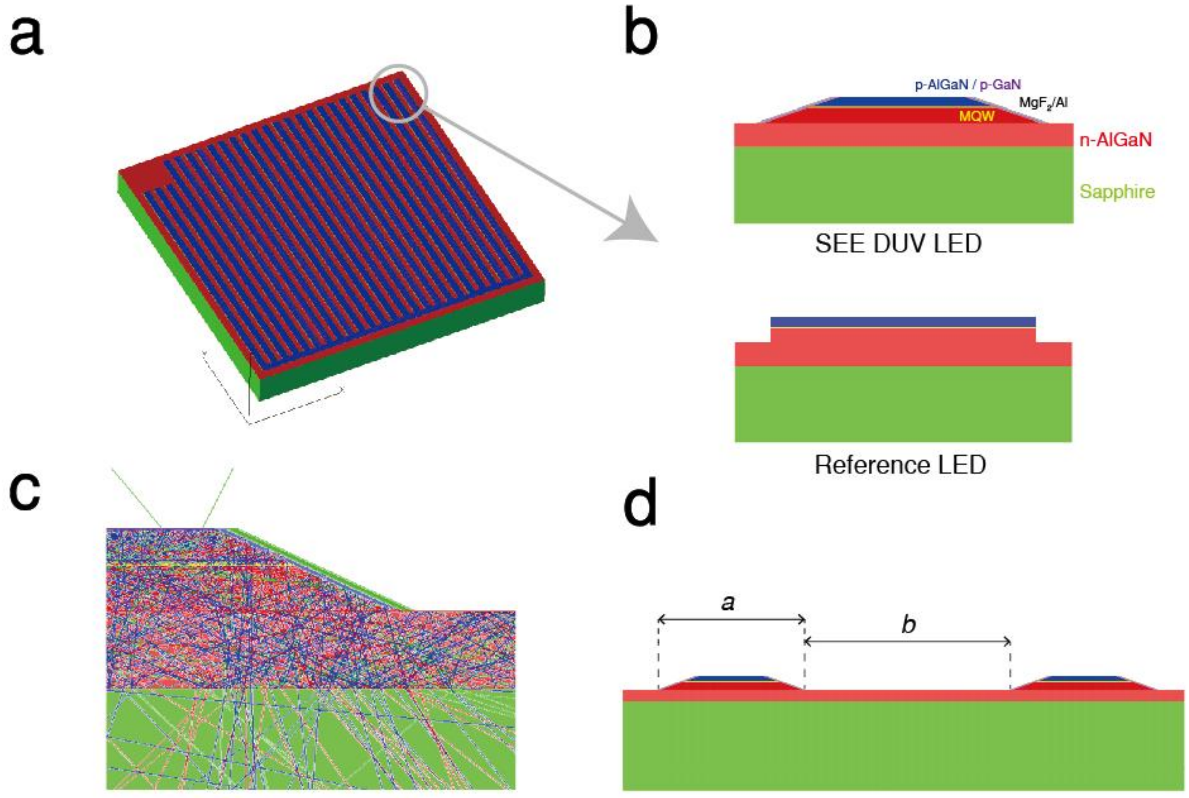

Supplementary Figure S5. Ray tracing simulations. (a) Schematic LED chip geometry for the SEE DUV LED having 25 stripes. (b) Schematic cross-sectional views of a mesa stripe for a SEE DUV LED and a reference LED. (c) An image showing the propagation of light rays in a SEE DUV LED during the ray tracing simulation. Large amount of light is trapped in the device because of total internal reflection. SEE structure can effectively extract photons heading toward sidewalls. (d) Width of mesa stripe *a* and distance between stripes *b*, which are summarized in Table S2.

Supplementary Table S2. Geometrical parameters of reference and SEE DUV LEDs used for the ray tracing simulations.

	Reference (5 stripes)	SEE DUV LED (5 stripes)	SEE DUV LED (25 stripes)	SEE DUV LED (45 stripes)
Chip size	1mm × 1mm	1mm × 1mm	1mm × 1mm	1mm × 1mm
a (μm)	179.6	179.6	25.52	8.4
b (μm)	13	13	13	13
Perimeter length (mm)	10.81	10.81	46.01	81.35

Supplementary Figure S6. Finite element method analysis

Numerical analysis using the finite element method (FEM) were performed by using a commercial FEM software package, COMSOL multiphysics, to calculate the electrical field distribution of DUV emission from various LED structures in order to check the feasibility of the SEE DUV LED concept. In the simulations, a modified Maxwell's equation, $\nabla \times (\nabla \times \boldsymbol{E}) - k_0^2 \varepsilon_r \boldsymbol{E} = 0$, where k_0 is the wave vector in vacuum, $\boldsymbol{E}$ is the electric field vector and ε_r is relative permittivity is solved. The absorption coefficients of the AlGaN and GaN layer at 275 nm are 10 cm^{-1} and 170,000 cm^{-1}, respectively[1,2]. The refractive indices used for GaN, AlGaN, MgF_2 and Al at 275nm are 2.61, 2.45, 1.4 and 0.216, respectively. The structures under FEM simulations include a 180 nm-thick p-GaN, a 2μm-thick AlGaN, and MgF_2/Al (250/150 nm). A reference LED with one stripe having vertical sidewalls and SEE DUV LEDs with one, two, three stripes having MgF_2/Al reflectors on the inclined sidewalls were simulated. The distribution of the electric field amplitude emitted from dipole sources located at the MQW active region is shown in supplementary Fig. S6a. Inspection of the Fig. reveals very strong bottom emission. Top emission is negligible compared to the bottom emission due to strong absorption in the p-GaN layer. The reference LED shows a strong emission through the vertical sidewalls which does not contribute in a significant way to the total light output. However, the strong sidewall emission can be effectively redirected to the sapphire substrate by the MgF_2/Al mirrors on the inclined mesa sidewalls in the SEE DUV LED, increasing the total light output. As the number of the stripe increases, the electrical field amplitude distribution below the substrate becomes stronger due to more effective reflection of the sidewall-directed emission with less absorption when travelling through the in-plane direction. Supplementary Fig. S6b shows the electric field amplitude of both TE and TM polarized light detected at the bottom. Both TE and TM polarized emissions are enhanced as the number of stripes increases. In particular, anisotropic TM polarized light shows a steeper enhancement than isotropic

TE polarized light when increasing number of stripes; this is because more anisotropic TM light is reflected at the sidewall reflectors than isotropic TE light.

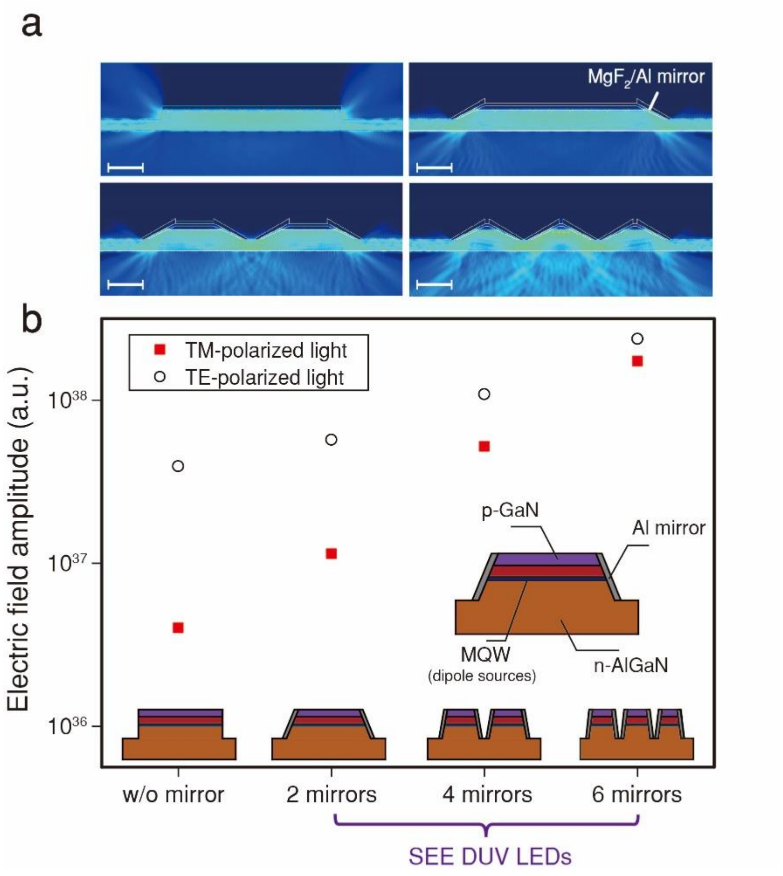

Supplementary Figure S6. Light extraction results using finite element method (FEM) analysis. (a) Electric field amplitude distribution of light emitted from the dipole sources. Each structure represents the reference, and SEE DUV LEDs with 2 mirrors, 4 mirrors, and 6 mirrors, respectively (scale bar, 2 μm). (b) Calculated TE and TM electric field amplitude for each structure shown by schematic illustration at bottom. The amplitude values are converted into log scale to show the wide range of amplitudes.

Supplementary Figure S7. Current-voltage characteristics of DUV LEDs

Representative current-voltage (I-V) characteristics of the reference and the SEE DUV LEDs having 5 and 45 stripes are shown in Supplementary Fig. S7. Typical rectifying curves have turn-on voltages near 4.4 V which is energy-equivalent to the 275 nm emission wavelength. As the number of stripes increases, the operating voltage becomes lower due to a larger n-contact area. With the same number of stripes, SEE DUV LEDs show a little bit higher operating voltage than the reference, presumably due to the reduced p-contact area and increased distance between p-contact and n-contact due to thermal reflow process; this can be solved by optimizing the fabrication process and photomask design.

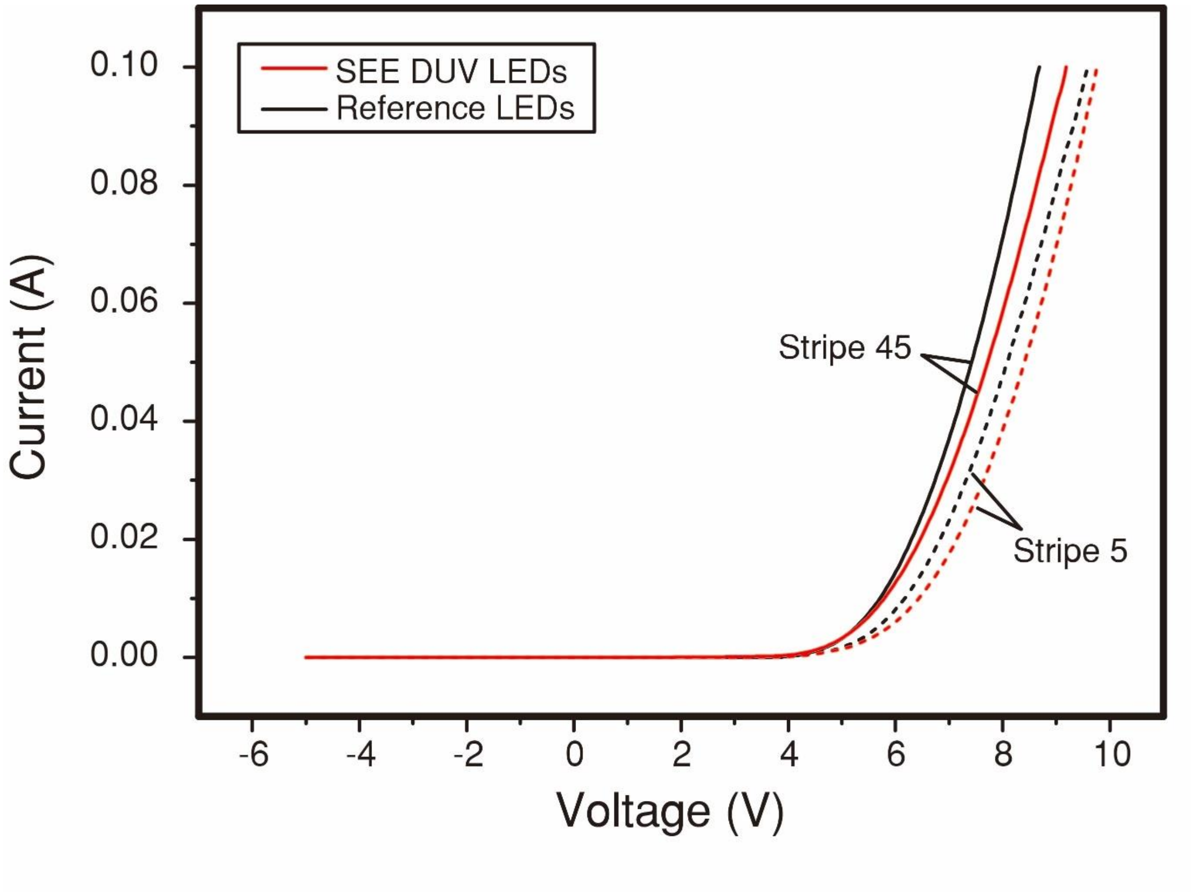

Supplementary Figure S7. Representative I-V characteristics of the reference and SEE DUV LEDs with 5 and 45 active mesa stripes.

Supplementary Figure S8. Estimation of relative LEE and IQE

Relative LEE and IQE values of both types of LEDs are obtained from the measured relative internal quantum efficiency (IQE) defined as photocurrent divided by injection current, and the light output power (LOP), by using the equation, $EQE = LOP/I = IQE \times LEE$. Although, the I-V characteristics of both LEDs are little bit different as shown in Figure S7 possibly due to difference in the area of mesas (SEE DUV LEDs have inclined sidewalls but reference LEDs do not) as well as in current spreading, which can make a small difference in the IQE, we believe that such difference in the IQE would not make a considerable effect on the estimation of *relative* LEE. Therefore we assume that all the DUV LEDs have the similar IQE value at the same injection current density (the LEDs are fabricated using the same epi-wafer), and since the LEE does not depend on the injection current density, the normalized IQE is inferred from a representative EQE curve. Since each structure with a different number of stripes has a different area of the active mesa, the injection current *density* is different for the same injection current. Due to the efficiency droop that is a function of the current density, normalized IQE values of LEDs with a different number of stripes can be estimated from the EQE curve. Supplementary Figure S8 shows the normalized IQE of a fabricated DUV LED as a function of injection current density. Normalized IQE values for the SEE DUV LEDs with various numbers of stripes, indicated by red dots, are extracted, and used for the estimation of the LEE of the reference and the SEE DUV LEDs, as shown in Fig. 3b. The inset Fig. shows a schematic of the measurement setup. LOP for all devices are measured at the bottom using a Si-based photodiode.

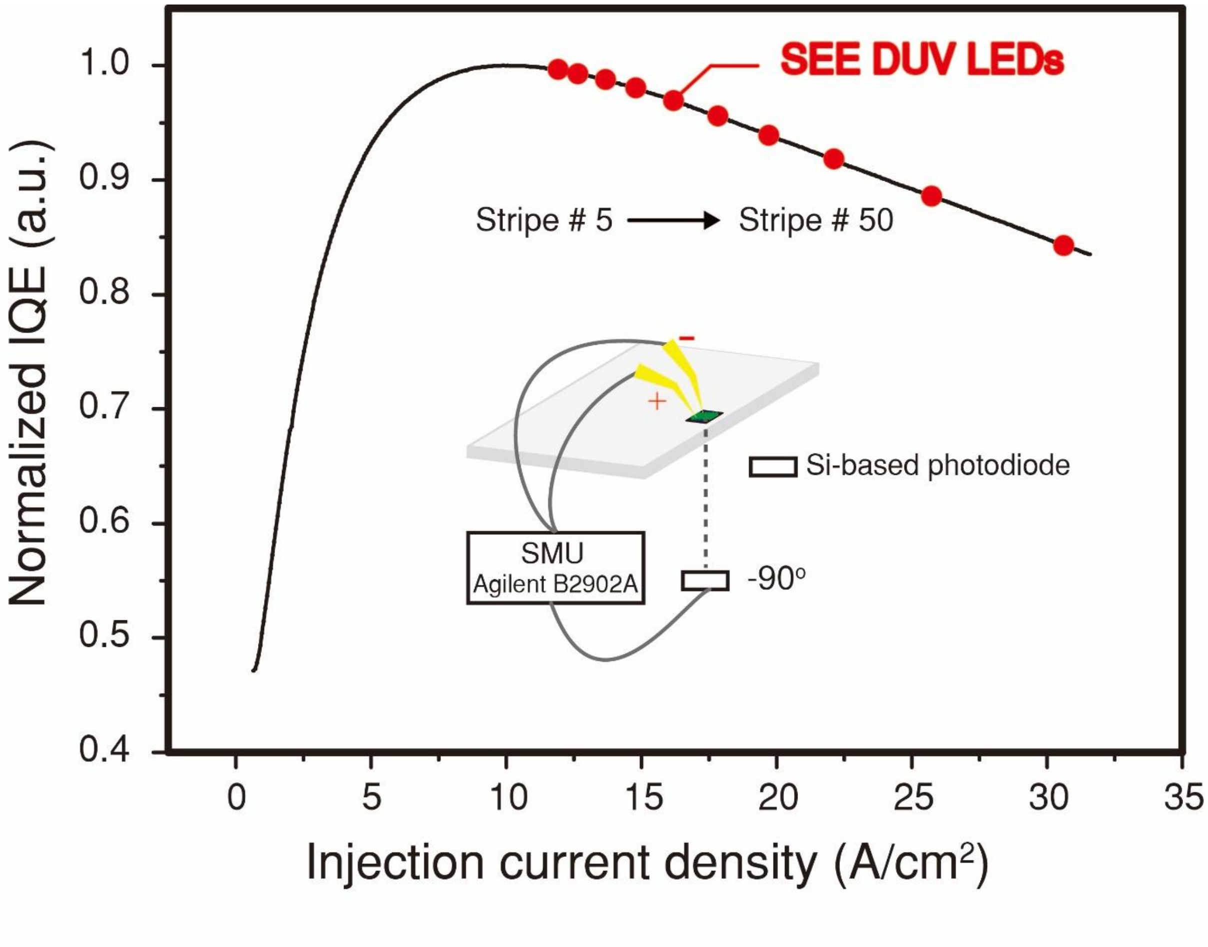

Supplementary Figure S8. Normalized IQE curve of a DUV LED fabricated using the same epi-wafer as the SEE DUV LEDs. Red dots indicate normalized IQE values of SEE DUV LEDs with different number of stripes, *i.e*. different areas of active mesa region.

Supplementary Information. **Estimation of the LEE by an analytic model**

We develop an analytic model to accurately estimate the LEE of AlGaN-based DUV LEDs by taking into account critical factors affecting the LEE including the TM/TE polarization ratio, the number of stripes, the current crowding effect, and the effect of interface roughening.

At first, the current crowding effect is considered and included in the model as a weighting factor. Due to the lateral carrier transport in the highly resistive AlGaN layers of the DUV LEDs grown on an insulating sapphire substrates, the injection current crowds near the edge of the active mesa stripes, resulting in an exponential decrease in LOP from the edge. Therefore, the current spreading length (L_s), defined as the length where the current density (J) drops to the 1/e value of the current density at the edge, is defined as[3],

$$L_s = \frac{\sqrt{(\rho_c + \rho_p t_p) \cdot t_n}}{\rho_n}, \quad (1)$$

(ρ_c is the p-type specific contact resistance, ρ_p is the resistivity of the p-type layer, ρ_n is the resistivity of the n-type cladding layer). With L_s, the current density as a function of distance from the edge of the active mesa stripe x is given by,

$$J(x) = J(0) \cdot exp(-\frac{1}{L_s} \cdot x) \quad (2)$$

In order to apply such a non-uniform current distribution along x into the estimation of the LEE, we introduce a weighting factor (WF) for the intensity of each dipole source. When the injection current is the same for all the devices, the following relation based on the integration by the rectangular method is valid.

$$\sum_{n=1}^{N+1} J(0) \cdot \exp\left(-\frac{1}{L_S} \cdot \frac{L_m}{N+1} \cdot n\right) \cdot \frac{L_m}{N+1} = C \quad (3)$$

where N is the number of dipole sources, L_m is the length of the one stripe, and C is a constant. The equation can be simplified by replacing $\frac{1}{L_S} \cdot \frac{L_m}{N+1}$ by k to obtain,

$$J(0) \cdot \frac{L_m}{N+1} \cdot \sum_{n=1}^{N+1} \exp(-n \cdot k) = C \quad (4)$$

$$J(0) \cdot \frac{L_m}{N+1} \cdot \frac{\exp(-k) \cdot [1 - \exp(-(N+1) \cdot k)]}{1 - \exp(-k)} = C \quad (5)$$

$$J(0) = C \cdot \left(\frac{L_m}{N+1} \cdot \frac{\exp(-k) \cdot [1 - \exp(-(N+1) \cdot k)]}{1 - \exp(-k)} \right)^{-1} \quad (6)$$

In the rectangular integration method, the area of one rectangle can be used as the weighting factor, *WF*(*n*), given as,

$$WF(n) = J(0) \cdot \exp(-n \cdot k) \cdot \frac{L_m}{N+1} \quad (7)$$

$$WF(n) = C \cdot \left(\frac{L_m}{N+1} \cdot \frac{\exp(-k) \cdot [1 - \exp(-(N+1) \cdot k)]}{1 - \exp(-k)} \right)^{-1} \cdot \exp(-n \cdot k) \cdot \frac{L_m}{N+1} \quad (8)$$

Next, we discuss the effective critical angle at the interface between two optical media. In our analytic model, there are two interfaces encountered by light travelling toward a bottom direction, the n-AlGaN/sapphire and sapphire/air interfaces. When the interfaces are planar, two critical angles should be considered for optical loss by the total internal reflection. The critical angle at the sapphire/air surface is

$$\theta_{c,sapphire/air} = sin^{-1}(\frac{n_{air}}{n_{Sapphire}}) \cong 22.6° \quad (9)$$

Similarly, the critical angle at the interface between n-AlGaN and sapphire substrate is

$$\theta_{c,nAlGaN/sapphire} = sin^{-1}(\frac{n_{Sapphire}}{n_{nAlGaN}}) \cong 43.8° \quad (10)$$

Considering the two planar interfaces, the effective critical angle $\theta_c{}^*$ is given as

$$\theta_c{}^*(planar) = sin^{-1}(\frac{n_{air}}{n_{nAlGaN}}) \quad (11)$$

It is well known that by roughening or texturing the interface, one can overcome the total internal loss, thus, increase the effective critical angle of total internal reflection at the interface, and consequently increase the LEE, which we consider in the analytic model. Introducing the surface roughening factor *SR*, the equation for the effective critical angle can be generalized as

$$\theta_c{}^* = sin^{-1}\left[\frac{n_{sapphire}}{n_{nAlGaN}}\cdot \sin\left\{(sin^{-1}\frac{n_{air}}{n_{sapphire}})\cdot SR\right\}\right] \quad (12)$$

The value of *SR* can be obtained by ray-tracing simulations or reported enhancement values by using roughening or patterning of the sapphire substrate. We assume that conventional surface roughening technique can make about 50% enhancement in LEE based on the references including *IEEE Electron Device Letters* **30,** 496-498 (2009), *Optics Express* **17,** 13747-13757 (2009), *Nanotechnology* **16,** 1844-1848 (2005), and *Journal of Display Technology* **9,** 324-332 (2013). The enhancement of LEE by surface roughening can be represented by the increase in the effective critical angle which makes 50% larger LEE in the analytic model. We note that exact percentage of enhancement, and exact value of increased critical angle by surface roughening can be affected by many factors, thus general values are not easy to be estimated, which, however, does not make a critical difference in the trend and the conclusion shown in Fig. 4d.

Finally, we discuss the LOP of extracted light through the sapphire substrate. For a dipole located in the (x_n, y_n) position, the emitted light path can be classified into 3 cases: i) toward the sapphire substrate side, ii) toward the p-GaN side, and iii) toward the MgF_2/Al mirror. The light path toward the left-hand side ($0.5\pi \leq \theta \leq 1.5\pi$) can be simply calculated based on the symmetry.

In the first case, the amount of directly extracted light toward bottom direction is described as,

$$LOP_{m,direct} = \sum_{n=1}^{N}\int_{-\pi/2}^{\theta_c{}^*}\{(I_{TE}(\theta)+I_{TM}(\theta)\}\cdot e^{-\alpha\cdot l(n,\theta)}\cdot WF(n)\,d\theta \quad (13)$$

where α is absorption coefficient of AlGaN, $l(n,\theta)$ is length of light path depending on dipole location, and $\theta_c{}^*$ is the effective critical angle.

In the second case, we assume that all of light is absorbed in the p-GaN layer, which is reasonable considering the thick p-GaN layer with a large absorption coefficient of GaN at 275 nm.

$$LOP_{m,p\_GaN} = \sum_{n=1}^{N}\int_{\gamma}^{90°}\{(I_{TE}(\theta)+I_{TM}(\theta)\}\cdot e^{-\alpha\cdot l(n,\theta)}\cdot WF(n)d\theta \cong 0 \quad (14)$$

In the third case, we need to consider the reflectance, *R*, of Al/MgF_2 ODR. β, γ, I_{TM} and I_{TE} are described in Fig. 4a. The values for γ and β also depend on the dipole location.

$$LOP_{m,reflected} = \sum_{n=1}^{N} \int_{-\beta}^{\gamma} \{(I_{TE}(\theta) + I_{TM}(\theta)\} \cdot e^{-\alpha \cdot l(n,\theta)} \cdot WF(n) \cdot Rd\theta \tag{15}$$

References

1. Yu, G. et al. Optical properties of wurtzite structure GaN on sapphire around fundamental absorption edge (0.78-4.77 eV) by spectroscopic ellipsometry and the optical transmission method. *Appl. Phys. Lett.* **70,** 3209 (1997).
2. Liu, Z., Wang, K., Luo, X. & Liu, S. Precise optical modeling of blue light-emitting diodes by Monte Carlo ray-tracing. *Opt. Express* **18,** 9398-9412 (2010).
3. Guo, X. & Schubert, E. F. Current crowding and optical saturation effects in GaInN/GaN light-emitting diodes grown on insulating substrate. *Appl. Phys. Lett.* **78,** 3337 (2001).
